# MazF activation promotes translational heterogeneity of the *grcA* mRNA in *Escherichia coli* populations

**DOI:** 10.7717/peerj.3830

**Published:** 2017-09-21

**Authors:** Nela Nikolic, Zrinka Didara, Isabella Moll

**Affiliations:** 1 Department of Microbiology, Immunobiology and Genetics, Max F. Perutz Laboratories, Vienna Biocenter (VBC), University of Vienna, Vienna, Austria; 2 Current affiliation: Institute of Science and Technology Austria (IST Austria), Klosterneuburg, Austria; 3 Current affiliation: Department of Life Sciences, IMC University of Applied Sciences Krems, Krems an der Donau, Austria

**Keywords:** Toxin-antitoxin system, *mazEF* module, Phenotypic heterogeneity, Gene expression, Fluorescent reporter, Flow cytometry

## Abstract

Bacteria adapt to adverse environmental conditions by altering gene expression patterns. Recently, a novel stress adaptation mechanism has been described that allows *Escherichia coli* to alter gene expression at the post-transcriptional level. The key player in this regulatory pathway is the endoribonuclease MazF, the toxin component of the toxin-antitoxin module *mazEF* that is triggered by various stressful conditions. In general, MazF degrades the majority of transcripts by cleaving at ACA sites, which results in the retardation of bacterial growth. Furthermore, MazF can process a small subset of mRNAs and render them leaderless by removing their ribosome binding site. MazF concomitantly modifies ribosomes, making them selective for the translation of leaderless mRNAs. In this study, we employed fluorescent reporter-systems to investigate *mazEF* expression during stressful conditions, and to infer consequences of the mRNA processing mediated by MazF on gene expression at the single-cell level. Our results suggest that *mazEF* transcription is maintained at low levels in single cells encountering adverse conditions, such as antibiotic stress or amino acid starvation. Moreover, using the *grcA* mRNA as a model for MazF-mediated mRNA processing, we found that MazF activation promotes heterogeneity in the *grcA* reporter expression, resulting in a subpopulation of cells with increased levels of GrcA reporter protein.

## Introduction

Bacteria frequently experience stressful conditions in their natural habitats, such as occurrence of toxins or depletion of nutrients. To cope with continuous changes in their environment, bacteria have evolved protection programs called the bacterial stress response (Hengge, 2011). Studies on the bacterial stress response have been mostly conducted at the level of bulk populations. However, a recent genome-wide screen has shown that genes involved in the stress response display particularly high levels of variation in gene expression, which was measured in clonal populations of *Escherichia coli* growing in homogeneous environmental conditions (Silander et al., 2012). Many similar studies have initiated a new area of research addressing the benefits of heterogeneous gene expression for bacterial populations. Taken together, these studies provide compelling evidence that phenotypic variation in clonal bacterial populations can be beneficial in the face of environmental fluctuations by granting individual cells a higher probability to survive (Kussell & Leibler, 2005; Acar, Mettetal & Van Oudenaarden, 2008; Arnoldini et al., 2014).

In general, the bacterial stress response is characterized by a profound alteration of the transcriptional program leading to the adaptation to the given conditions (Hengge, 2011). Furthermore, bacteria can adjust to environmental constraints at the post-transcriptional level. One such mechanism has recently been described (Vesper et al., 2011): When *E. coli* populations encounter stress, a subset of mRNAs is processed upstream of the AUG start codon, resulting in the truncated 5′-untranslated region (UTR) and/or removal of their Shine-Dalgarno sequence (Sauert et al., 2016). In addition, ribosomes are modified and specifically translate these processed mRNAs; the 3′-end of the 16S rRNA is removed from the ribosome, thereby forming specialized stress-ribosomes that lack the anti-Shine-Dalgarno sequence (Vesper et al., 2011). Both mechanisms are mediated by the endoribonuclease MazF, the toxin component of the toxin-antitoxin (TA) system *mazEF*, which is a type II TA locus (Gerdes, Christensen & Løbner-Olesen, 2005; Gerdes & Maisonneuve, 2012).

TA loci are generally widespread in bacterial and archaeal genomes (Gerdes, Christensen & Løbner-Olesen, 2005; Van Melderen, 2010). Stress-induced toxin activation inhibits basic cellular processes, such as replication, translation and peptidoglycan synthesis. There are at least five different types of TA systems, which are categorized depending on the mode of action of the antitoxin (Goeders & Van Melderen, 2014). For example, the antitoxin MazE of the above mentioned TA system *mazEF* is a short-lived protein that neutralizes toxin expression and activity by direct binding to the toxin MazF, classifying it as a type II TA system. Besides chromosomally encoded TA systems, plasmid-encoded TA systems are likewise encoded on naturally occurring plasmids, such as the F plasmid or the R1/R100 plasmids (Gerdes, Christensen & Løbner-Olesen, 2005; Van Melderen, 2010; Ramisetty & Santhosh, 2017). These plasmid-encoded TA systems ensure plasmid maintenance in growing bacterial populations by killing those cells that do not inherit the plasmid. Even though the physiological role of plasmid-encoded TA systems is well established (by the mechanism of ‘post-segregational killing’), possible physiological roles of chromosomally encoded TA systems still remain elusive, and so far include growth modulation, persistence after exposure to antibiotics or other stress conditions, regulation of gene expression, and protection against invading plasmids and phages (Gerdes, Christensen & Løbner-Olesen, 2005; Van Melderen, 2010; Gerdes & Maisonneuve, 2012; Ramisetty & Santhosh, 2017).

Several recent studies implicated TA systems in the emergence of phenotypic heterogeneity in bacterial populations, exhibited as variation in gene expression (Maisonneuve, Castro-Camargo & Gerdes, 2013), cell size (Kasari et al., 2010) or growth rate (Klumpp & Hwa, 2014), as well as persister cell formation (Balaban et al., 2004; Maisonneuve et al., 2011). Bacterial persistence is one of the most studied functions of phenotypic heterogeneity that arises in populations of genetically identical cells independently of genetic or environmental differences. However, persister cells represent a minor fraction of a clonal population that can endure various antibiotic treatments (Balaban et al., 2004). It has been shown that MazF overproduction results in a higher number of persister cells upon treatment with different antibiotics (Maisonneuve et al., 2011).

The TA locus *mazEF* is transcribed into a polycistronic mRNA, where *mazE* encodes the short-lived antitoxin and *mazF* encodes for the stable toxin that cleaves single-stranded RNA regions at ACA-sequences in *E. coli* (Zhang et al., 2003). During periods without stress MazE forms a stable complex with MazF, thereby inhibiting the endoribonucleolytic activity of MazF (Kamada, Hanaoka & Burley, 2003). Moreover, besides MazE alone, the MazE-MazF complex can repress transcription of the operon (Marianovsky et al., 2001). When bacteria encounter stress, such as starvation, DNA damage, heat shock, and treatment with antibiotics, as for instance rifampicin, chloramphenicol and spectinomycin (Sat et al., 2001; Christensen et al., 2003; Hazan, Sat & Engelberg-Kulka, 2004), the Lon (Maisonneuve et al., 2011) and ClpAP proteases (Aizenman, Engelberg-Kulka & Glaser, 1996) degrade the antitoxin MazE, and thus enable MazF to exert its endoribonucleolytic activity.

One typical transcript identified to be processed by MazF within the 5′-UTR is the *grcA* mRNA (formerly called *yfiD*) encoding protein GrcA that has been shown to reactivate an oxidatively damaged pyruvate formate-lyase (Wagner et al., 2001). The transcript comprises two ACA sites closely upstream of the start codon, which are cleaved by MazF during stress (Vesper et al., 2011; Sauert et al., 2016). Consequently, the processed transcript is selectively translated by the MazF-modified ribosomes lacking the 3′-terminus of the 16S rRNA (Vesper et al., 2011; Sauert et al., 2016).

Here, we aimed to address the questions: (1) how transcription of the *mazEF* module differs between single cells in a population, and (2) how the MazF-mediated processing of distinct mRNAs affects protein synthesis at the single-cell level during stress. To this end, we followed the induction of *mazEF* transcription by different stressors using the chromosomally integrated transcriptional reporter *P*_*maz*_-*gfp* in single bacterial cells, and measured the increase in GFP fluorescence by flow cytometry. Moreover, we employed a chromosomally integrated *gfp* reporter fusion comprising the transcriptional and translational regulatory regions of the *grcA* gene. These analyses revealed that MazF-mediated processing of the *grcA-gfp* mRNA consequently promotes heterogeneity in the *grcA* reporter gene expression in clonal populations. Taken together, our results suggest that the MazF concentration is kept at low levels during adverse conditions, but can considerably shape the gene expression profile of a cell.

## Material and Methods

### Bacterial strains and plasmids

We used derivatives of *E. coli* strain K-12 MG1655 (Blattner et al., 1997; Müller et al., 2016) when addressing effects of antibiotic treatments and amino acid starvation, and derivatives of *E. coli* strain BW27784 containing a native copy of the *mazEF* operon (Khlebnikov et al., 2001) for experiments with arabinose-inducible systems. The open reading frame of the employed reporter gene Em*gfp*ΔACA is devoid of ACA sites (Sauert, 2015; Oron-Gottesman et al., 2016), whilst the amino acid sequence corresponds to the wild-type fast-folding Emerald GFP (Tsien, 1998). The chromosomally integrated *mCherry* reporter (Cox III, Dunlop & Elowitz, 2010) under control of the phage *λ* promoter *P*_R_ is used to infer constitutive gene expression. All strains and plasmids are listed in Table S1.

All primers used in this study are listed in File S1, section ‘Sequences’. Briefly, the translational *grcA*_*wt*_ reporter was constructed by fusing the fragment of the *grcA* gene comprising nucleotides −278 to +45 in frame to the *gfp* coding sequence *via* a linker encoding three glycine residues. The *grcA*_*ATA*_ reporter was constructed by fusion-PCR, modifying C to T in the sequence of the *grcA*_*wt*_ reporter at positions −1 and −32 upstream of the start codon. Both *grcA* reporters were cloned into a modified CRIM plasmid pAH68-frt-chlor (kindly provided by T. Bergmiller; Haldimann & Wanner, 2001) *via* EcoRI/PstI restriction sites. The *grcA*_*wt*_ reporter was further cloned into a low copy plasmid pZS*12-GFP (kindly provided by C. Guet) *via* XhoI/HindIII restriction sites. The transcriptional reporter *P*_*maz*_-*gfp* was cloned into a modified CRIM plasmid pAH120-frt-chlor (kindly provided by T. Bergmiller; Haldimann & Wanner, 2001), and the promoter sequence, i.e., intergenic region between *relA* and *mazE*, was flanked with XhoI/BamHI restriction sites. All constructs were inserted into neutral phage attachment sites in the *E. coli* genome using the CRIM system: the plasmids pAH68-frt-chlor and pAH120-frt-chlor were integrated into *att*HK022 and *attλ*, respectively (Haldimann & Wanner, 2001). After integration, the chloramphenicol resistance marker was removed by using the site-specific Flp-recombinase (Cherepanov & Wackernagel, 1995).

### Growth conditions

Rich, defined media was composed of 1× M9 salts, 1 mM MgSO_4_, 0.1 mM CaCl_2_, 0.5% casamino acids (Fluka), and 10 mM maltose. Clones were first streaked from frozen glycerol stocks on LB (Lennox modification) agar to obtain single colonies. A single colony was inoculated overnight for 15–16 h, in 4 ml of media, at 37 °C with shaking at 165 rpm. Bacterial growth was monitored by measuring the optical density at 600 nm (OD_600_) with a spectrophotometer. The overnight cultures were diluted 1 to 1,000 into fresh media, OD_600_ ∼  0.007, and grown for 2 h 15 min. The exponential cultures were then split in two or more flasks; with one flask serving as a control, while different stressors (or arabinose to induce *mazF* expression) were added to other flasks. After 3–4.5 h of stress, 4 ml of stressed cultures were washed with 1×  PBS, resuspended in 4 ml of pre-warmed fresh media and allowed to regrowth. In general, the cultures were analyzed by flow cytometry at three time points: (t1) 2–3 h after stress induction; (t2) 5.5–7 h after stress induction, or recovered for 1.5–2.5 h after initial 3–4.5 h under stress; (t3) 21 h after stress induction, or recovered for 15.5–18 h after initial 3–4.5 h under stress.

### Flow cytometry

#### Flow cytometers

Analysis was performed with FACS Calibur for GFP reporter strains and LSR Fortessa for mCherry-GFP systems (BD, San Jose, California, USA). Calibur is equipped with an argon laser with excitation at 488 nm. In total 100,000 or 150,000 events were acquired for each sample at low speed, with the following settings –FSC-H (forward scatter): E01, SSC-H (side scatter): 349 V, FL1-H: 813 V; log mode; primary threshold on SSC. Fortessa is equipped with lasers with excitation at 488 nm and 561 nm, and filters for detection of GFP fluorescence (BP filter 530/30 nm) and mCherry fluorescence (BP filter 610/20 nm). For each sample 50,000 events were acquired at low speed, with the following settings –FSC-H (forward scatter): 380 V, SSC-H (side scatter): 220 V, FITC-H: 430 V, PE-Texas Red-H: 590 V; log mode; compensation FITC-PE Texas Red 0.2; threshold: SSC at 200 V and FSC at 200 V.

#### Data analysis and visualization

**Figure 1:**
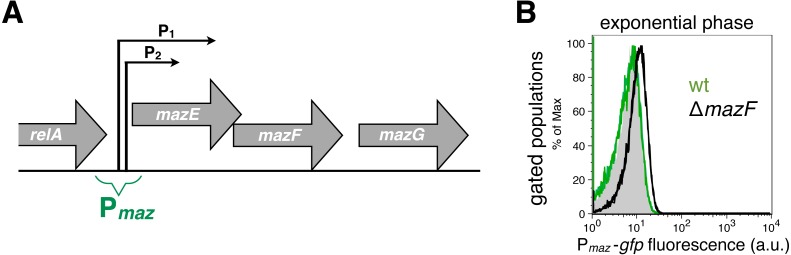
Strong and weak repression of the *mazEF* transcription. (A) A scheme of the *mazEFG* operon (Keseler et al., 2013). The transcriptional reporter *P*_*maz*_-*gfp* includes intergenic region between the *relA* and *mazE* genes together with promoters *P*_*maz*1_ (*P*_1_) and *P*_*maz*2_ (*P*_2_), initially named *P*_2_ and *P*_3_, respectively. The promoters are located 13 nt apart, and the upstream promoter is 10 times more active than the downstream promoter (Marianovsky et al., 2001). Details of the reporter construction are given in File S1. (B) We used the reporter *P*_*maz*_-*gfp* to quantify weak repression of the *mazEF* transcription by comparing the GFP signal in the Δ*mazF* strain (black histogram, strain NN229) and the isogenic wild-type strain (green histogram; strain NN227), measured in the exponential phase. The GFP fluorescence level of the wild-type strain NN227 was comparable to the fluorescence of the reporterless MG1655 strain (grey shaded histogram), thus corresponding to bacterial autofluorescence (see File S1 for Source Data).

Raw fcs files were exported from FlowJo (Tree Star, Inc.) into a custom R script, similar to the script published as Dataset S2 in Silander et al. (2012), with R commands for gating indicated in File S1, section ‘R commands’. Flow cytometry plots were made in FlowJo. All events were analyzed for the experiments, unless indicated otherwise. For the experiment presented in Fig. 1, in total 10,000 cells were analyzed with R script after filtering (gating) events on the basis of FSC and SSC measurements (size parameters), and visualized by using auto-gating tool on the size parameters in FlowJo.

**Figure 2:**
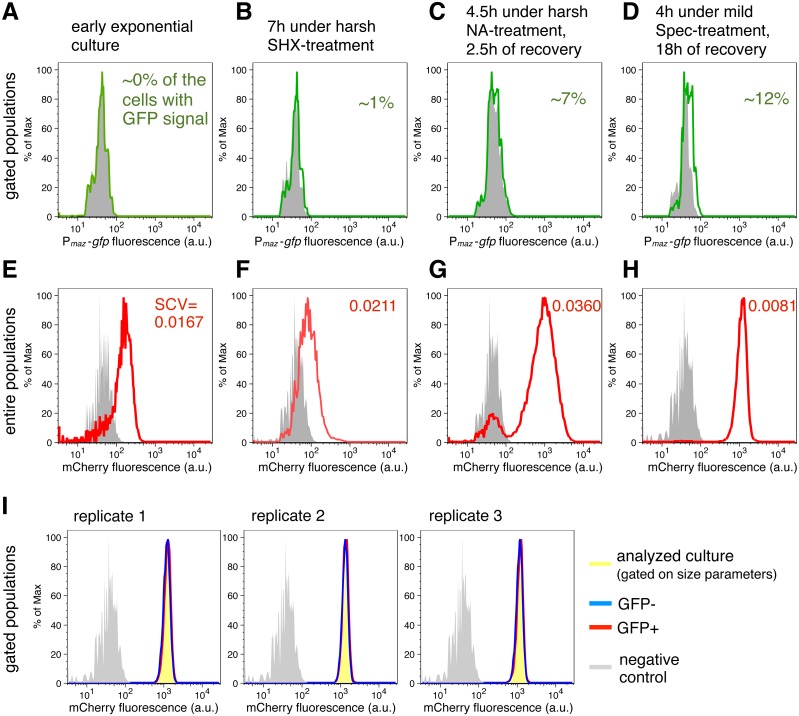
Transcriptional de-repression and activation of the *mazEF* module, and constitutive gene expression under different conditions. (A–D) GFP fluorescence of strain NN230 harboring the *P*_*maz*_-*gfp* reporter is depicted in green; background GFP fluorescence of strain TB205 is depicted in grey. We measured the GFP signal in the cells gated on the size parameters and mCherry signal, to exclude filaments, dead cells and other debris that can influence the calculations. Analysis of the expression of the *P*_*maz*_-*gfp* reporter and the constitutive *mCherry* reporter upon amino acid starvation induced with serine hydroxamate, as well as upon antibiotic treatments—namely ampicillin, chloramphenicol, nalidixic acid, spectinomycin—are reported in Table 1 for ‘harsh’ conditions and in Table 2 for ‘mild’ conditions. (E–H) mCherry fluorescence of strain NN230 harboring the *λP*_*R*_-*mCherry* reporter is depicted in red; background mCherry fluorescence of strain MG1655 is depicted in grey. To infer population heterogeneity, we analyzed squared coefficient of variation (SCV; defined as squared standard deviation divided by the squared mean) in the mCherry fluorescence of the entire populations, without gating. (I) We first gated populations based on the size parameters, to minimize influence of cell size on the level of GFP fluorescence. Then, we analyzed the constitutive mCherry fluorescence in the fraction of cells without (GFP−) or with (GFP+) GFP signal of strain NN230, recovered after mild Spec-treatment. The same GFP threshold was applied in all replicates gated on approximately 20,000 cells by using FlowJo, and then two fractions per replicate, GFP- and GFP+ fraction, were analyzed in R. There is no correlation between GFP signal and mCherry signal in the analyzed replicates. For replicate 1: Spearman’s *rho* = 0.104, *p* = 5.7*E* − 50, *N* = 20,440 events; for replicate 2: *rho* = 0.088, *p* = 2.0*E* − 36, *N* = 20,546 events; for replicate 3: *rho* = 0.072, *p* = 2.5*E* − 25, *N* = 20,542 events.

Cytometry setup and analysis for Fig. 2 was as follows. The cultures were analyzed after approximately 3 h (time point t1), 7 h (time point t2) and 23 h (time point t3). We used concentrations of stressors above their minimum inhibitory concentration (MIC), referred to as ‘harsh’ conditions (Table 1), and below MIC referred to as ‘mild’ conditions (Table 2) (Tosa & Pizer, 1971; Sulavik et al., 2001; Chaudhuri et al., 2010): ampicillin (Amp) 100 µg/ml and 1 µg/ml, chloramphenicol (Cam) 15 µg/ml and 1 µg/ml, nalidixic acid (NA) 50 µg/ml and 1 µg/ml, spectinomycin (Spec) 90 µg/ml and 1 µg/ml, and serine hydroxamate (SHX, Sigma) 100 µg/ml and 1 µg/ml, as ‘harsh’ and ‘mild’ conditions, respectively.

**Table 1:** Expression of the *P*_*maz*_-*gfp* reporter in the presence of stressors at concentrations above the MIC levels (‘harsh’ stress conditions). Percentage of the cells that express the transcriptional reporter *P*_*maz*_-*gfp* above background level, measured before, during and after experiencing stress.

Time point			Percentage of the cells with GFP signal^a^ (gated populations)	SCV of log mCherry fluorescence (a.u.)^a^ (entire populations)	∼OD
t0	Exponential		∼0	0.0140 ± 0.001	0.23
t1	5 h of growth	Control	∼0	0.0156 ± 0.002	2.10
	3 h under stress	^c^Amp, 100 µg/ml	∼0	0.0327 ± 0.002	0.07
		Cam, 15 µg/ml	∼0	0.0176 ± 0.002	0.35
		NA, 50 µg/ml	∼0	0.0273 ± 0.005	0.68
		SHX, 100 µg/ml	0.78 ± 0.56	0.0157 ± 0.001	0.56
		Spec, 90 µg/ml	1.05 ± 0.35	0.0142 ± 0.002	0.42
t2	9 h of growth	Control	0.39 ± 0.07	0.0210 ± 0.002	7.16
	7 h under stress	Amp, 100 µg/ml	∼0	0.0265 ± 0.001	0.07
		Cam, 15 µg/ml	0.18 ± 0.11	0.0227 ± 0.004	0.56
		NA, 50 µg/ml	∼0	0.0315 ± 0.008	0.77
		SHX, 100 µg/ml	^b^**1.01 ± 0.19**	**0.0211 ± 0.004**	3.00
		Spec, 90 µg/ml	2.35 ± 0.46	0.0110 ± 0.002	0.62
	4.5 h under stress,	Regrowth, Amp-treated	0.02 ± 0.01	0.0300 ± 0.001	0.23
	then 2.5 h of regrowth	Regrowth, Cam-treated	5.41 ± 4.32	0.0148 ± 0.001	1.40
		Regrowth, NA-treated	**7.01 ± 2.94**	**0.0360 ± 0.000**	0.77
		Regrowth, SHX-treated	0.61 ± 0.05	0.0165 ± 0.001	4.21
		Regrowth, Spec-treated	1.31 ± 0.94	0.0171 ± 0.002	1.34
t3	25 h of growth	Control	1.13 ± 0.02	0.0075 ± 0.001	7.27
	4.5 h under stress,	Regrowth, Amp-treated	1.99 ± 0.38	0.0123 ± 0.001	3.85
	then 15.5 h of regrowth	Regrowth, Cam-treated	4.56 ± 0.38	0.0113 ± 0.001	7.81
		Regrowth, NA-treated	2.58 ± 0.75	0.0167 ± 0.001	6.71
		Regrowth, SHX-treated	4.04 ± 0.32	0.0147 ± 0.004	6.68
		Regrowth, Spec-treated	5.65 ± 0.92	0.0091 ± 0.001	8.62

**Notes.**

a (mean ±  standard error of the mean).

b Values in bold font are the examples depicted in Fig. 2.

c Amp, ampicillin; Cam, chloramphenicol; NA, nalidixic acid; SHX, serine hydroxamate; Spec, spectinomycin.

**Table 2:** Expression of the *P*_*maz*_-*gfp* reporter in the presence of stressors at concentrations below MIC levels (‘mild’ stress conditions). Percentage of the cells that express the transcriptional reporter *P*_*maz*_-*gfp* above background level, measured before, during and after experiencing mild stress conditions.

Time point			Percentage of the cells with GFP signal^a^ (gated populations)	SCV of log mCherry fluorescence (a.u.)^a^ (entire populations)	∼OD
t0	Exponential		^b^**∼0**	**0.0167 ± 0.002**	0.22
t1	5 h of growth	Control	∼0	0.0157 ± 0.000	1.34
	3 h under stress	^c^Amp, 1 µg/ml	3.13 ± 2.21	0.0159 ± 0.000	1.25
		Cam, 1 µg/ml	0.95 ± 0.34	0.0163 ± 0.003	0.64
		NA, 1 µg/ml	2.96 ± 1.26	0.0113 ± 0.002	1.15
		SHX, 5 µg/ml	2.36 ± 1.58	0.0173 ± 0.000	1.17
		Spec, 1 µg/ml	2.01 ± 1.14	0.0125 ± 0.002	1.08
t2	9 h of growth	Control	1.02 ± 0.46	0.0211 ± 0.003	5.93
	7 h under stress	Amp, 1 µg/ml	1.98 ± 0.51	0.0162 ± 0.000	5.67
		Cam, 1 µg/ml	0.29 ± 0.13	0.0188 ± 0.000	3.00
		NA, 1 µg/ml	1.22 ± 0.18	0.0185 ± 0.001	4.72
		SHX, 5 µg/ml	1.14 ± 0.29	0.0173 ± 0.000	5.13
		Spec, 1 µg/ml	1.65 ± 0.57	0.0173 ± 0.001	4.17
	4 h under stress,	Regrowth, Amp-treated	1.92 ± 0.08	0.0117 ± 0.001	5.88
	then 2.5 h of regrowth	Regrowth, Cam-treated	0.16 ± 0.06	0.0182 ± 0.000	3.48
		Regrowth, NA-treated	0.90 ± 0.04	0.0155 ± 0.000	5.34
		Regrowth, SHX-treated	1.84 ± 0.37	0.0111 ± 0.001	7.33
		Regrowth, Spec-treated	0.96 ± 0.18	0.0160 ± 0.000	5.37
t3	25 h of growth	Control	4.94 ± 1.77	0.0088 ± 0.001	7.83
	4 h under stress,	Regrowth, Amp-treated	3.90 ± 0.12	0.0072 ± 0.000	9.45
	then 18 h of regrowth	Regrowth, Cam-treated	2.18 ± 0.12	0.0118 ± 0.002	7.99
		Regrowth, NA-treated	11.69 ± 0.80	0.0094 ± 0.001	9.24
		Regrowth, SHX-treated	2.95 ± 0.24	0.0163 ± 0.006	9.99
		Regrowth, Spec-treated	**12.04 ± 1.96**	**0.0081 ± 0.001**	9.51

**Notes.**

a (mean ±  standard error of the mean).

b Values in bold font are the examples depicted in Fig. 2.

c Amp, ampicillin; Cam, chloramphenicol; NA, nalidixic acid; SHX, serine hydroxamate; Spec, spectinomycin.

In total, 50,000 events were acquired by the flow cytometer Fortessa for strain NN230. Minimally 20,000 events were processed per each sample, by filtering on the basis of FSC and SSC measurements (size parameters). The gating captured cells in the densest part of the FSC-SSC plot and therefore the gates consisted of cells with similar size and physiology due to the narrow range of FSC and SSC, respectively. Such analysis allows one to minimize the variation in fluorescence due to different cell size or cell damage, differences in stage of cell cycle, etc. Of those gated events, we only considered events that contained a mCherry signal above background level. The mCherry background level is defined as the 99th percentile of the measurements for the strain that does not contain the *mCherry* fluorescent reporter gene, which corresponds to autofluorescence of the MG1655 strain. This was one way how we could determine that the cells were alive or at least their membranes were not damaged during stress. For instance, cells damaged or lysed due to Amp-treatment could still be detected by flow cytometry within defined FSC and SSC parameters, but a constitutive mCherry signal would not be detected, and therefore we do not include those dead cells into further analysis.

The GFP background level is defined as the 99th percentile of measurements for the strain that does not contain the *gfp* fluorescent reporter gene (which corresponds to autofluorescence of the TB205 strain; Bergmiller et al., 2017), averaged over all conditions. These subsets were used for the subsequent analysis to calculate the number of cells that expressed *gfp* above background level. Finally, the number of GFP-positive cells was corrected with the number of cells with GFP fluorescence above background level measured in reporterless strains in the same condition, to eliminate false-positive GFP signals.

### Microscopy and image analysis

An exponentially growing culture of strain NN207 was divided into two flasks, and 0.2% Ara was added to one flask to induce *mazF* expression. Samples were taken from untreated cultures and cultures treated for 3 h and 22 h with Ara. The cells were fixed with 1% formaldehyde in 1× PBS, incubated for 2 h in the dark at room temperature using a nutator, followed by two washing steps with 1× PBS. The samples were kept in dark at 4 °C until microscopy the following day. 1.2 µl of a fixed bacterial sample was applied on a 1.5% agarose pad using a cavity slide as previously described (Bergmiller et al., 2011). Images were acquired with Olympus IX-81 inverse widefield epifluorescence microscope using objective UPlanSApo 100×/1.4 Oil, and Hamamatsu ORCA-ER detection system. The lamp intensity was set on 100%; exposure time of 130 ms for phase contrast images; and exposure time of 200 ms (filter TexasRed U-MWG, Ex. 510–550 nm, Em. LP 590 nm) for mCherry images. Cells were analyzed with the Matlab-based package *Schnitzcells* (Young et al., 2011), and segmented using mCherry fluorescence images.

### Statistical analysis

Statistics was done in SPSS, *R* and Microsoft Excel. Error bars in all graphs depict standard deviation from N replicates (independent cultures); N is indicated in every Figure Legend. We log_10_-transformed fluorescence data prior to analysis. The squared coefficient of variation (SCV) was used as a measure of variation in the reporter fluorescence. SCV is the squared value of coefficient of variation (CV), and CV is defined as standard deviation divided by the mean. We computed Pearson’s second skewness coefficient (median skewness) as skewness = 3*(mean − median)/standard deviation; and kurtosis type 2 in *R*. We used 2-tailed, heteroscedastic Student’s *t*-test to infer differences between two datasets for each measurement: between the fluorescence values of the *grcA*_*wt*_ and *grcA*_*ATA*_ reporters, and between the fluorescence values of the strain harboring P_*maz*_-*gfp* reporter (NN230) and the strain without *gfp* reporter gene (TB205). We used One-way ANOVA with Post Hoc Test Bonferroni to infer differences in four datasets concomitantly (fluorescence measurements of strains NN200, NN211, NN208, NN221). We used non-parametric Spearman’s two-tailed test to assess correlations between mCherry and GFP signals.

## Results

### Monitoring repression and de-repression of the *mazEF* transcription using the transcriptional reporter *P*_*maz*_-*gfp*

Expression of the *mazEF* module is negatively autoregulated, and the *mazEF* transcription from promoters *P*_*maz*__1_ and *P*_*maz*__2_ is strongly repressed under non-stressful conditions by the MazE protein or the MazE-MazF complex (Marianovsky et al., 2001; Keseler et al., 2013). However, repression by the MazE antitoxin alone is reduced by approximately 40% when compared to the MazE-MazF complex, as determined in the *E. coli* strain MC4100 (Marianovsky et al., 2001). In contrast, stressful conditions promote degradation of the MazE antitoxin (Aizenman, Engelberg-Kulka & Glaser, 1996; Maisonneuve et al., 2011), which subsequently results in de-repression of the *mazEF* transcription. Thus, the state of *mazEF* transcription depends on the ratio between toxin T and antitoxin A within a cell: weak repression (when T ≪ A), repression (when T ≈ A), or de-repression (when T ≫ A). This complex mechanism of transcriptional autoregulation of TA systems is generally termed ‘conditional cooperativity’ (Cataudella et al., 2013; Gelens et al., 2013).

Here, we used the transcriptional reporter *P*_*maz*_-*gfp* to indirectly monitor the regulation of the *mazEF* transcription in the *E. coli* strain MG1655 via GFP fluorescence (Fig.  1A). As a proof-of-principle for using this fluorescent reporter to infer the conditional cooperativity mechanism, we first quantified the activity of the *P*_*maz*_ promoters in the presence and absence of the MazF toxin. To this end, we calculated the difference of the mean levels of the GFP signal between the strain MG1655 Δ*mazF* and the isogenic wild-type strain, divided by the signal obtained with the wild-type strain (see File S1 for Source Data). Our data show that in the early exponential phase the mean GFP fluorescence level is increased by 60% in the Δ*mazF* strain relative to the wild-type strain (Fig. 1B), what can be attributed to the weak repression of transcription in the absence of MazF, i.e., when [MazF] ≪ [MazE].

### During and after exposure to antibiotics or amino acid starvation *mazEF* is transcribed at low levels

Next, we aimed to quantify *mazF* expression levels during adverse conditions that were reported to activate MazF using the transcriptional reporter *P*_*maz*_-*gfp*. Concomitantly, we determined the degree of cell-to-cell heterogeneity under these conditions using the constitutive *mCherry* reporter (Fig. 2). We measured the GFP fluorescence by flow cytometry upon treatment with ‘mild’ (below MIC values) and ‘harsh’ (above MIC values) concentrations of ampicillin (Amp), chloramphenicol (Cam), nalidixic acid (NA), or spectinomycin (Spec), as well as upon mimicking amino acid starvation by adding serine hydroxamate (SHX) (Table 1 for ‘harsh’ and Table 2 for ‘mild’ stress). To calculate the percentage of cells that display GFP signals above background level, we first gated the populations based on the size parameters, and second, based on a constitutively expressed *mCherry*. Such gating allowed us to analyze live cells with similar size, as described in details in ‘Material and Methods’.

Our analysis using the transcriptional reporter *P*_*maz*_-*gfp* revealed that GFP fluorescence was not detectable in the exponential phase (Fig. 2A). However, during SHX-treatment (Fig. 2B) and antibiotic treatments (Tables 1 and 2) the obtained GFP signals exceeded background levels. GFP fluorescence was generally lower during ‘harsh’ than during ‘mild’ stress because the high concentrations of the stressors either killed a fraction of the stressed populations or severely inhibited growth (OD_600_ values are given in Tables 1 and 2). Severe growth retardation implies reduction in overall translational capacity of cells, including decreased GFP synthesis. However, increased GFP signals were measured in all recovered cultures after removal of the stressor (Fig. 2C) when translation of the reporter *gfp* mRNA was no longer inhibited by the stressor. The maximal GFP signal was observed during recovery after treatment with 1 µg/ml Spec (Fig. 2D), i.e., about 12% of the cells expressed *gfp* above background level. We also included an additional statistical test indicating significantly higher mean levels of GFP fluorescence for the strain harboring *P*_*maz*_-*gfp* reporter compared to the strain without *gfp* reporter gene (*t*-test, *p* < 0.05), during stress and in the recovery periods (all *p*-values in File S1). These results suggest that during antibiotic treatment and amino acid starvation, and following stress removal, de-repression of the *mazEF* transcription occurs at low levels. Taken together, transcription and therefore *de novo* production of MazF is maintained at low levels during stress.

Besides determining the GFP fluorescence, we concomitantly monitored the expression of the reporter gene *λP*_R_-*mCherry* to infer constitutive expression (Fig. 2E), as the *λ* promoter *P*_R_ is specifically regulated only in the presence of the phage *λ* proteins (Berthoumieux et al., 2013). Given that expression of bacterial genes is regulated both through specific factors, such as transcription factors and post-transcriptional regulators, and global factors, such as the number of available RNA polymerases and ribosomes (Berthoumieux et al., 2013; Gerosa et al., 2013), the constitutive *mCherry* expression, which is solely affected by global factors, provides an estimate for the influence of the growth rate on gene expression in single cells (Klumpp, Zhang & Hwa, 2009; Scott et al., 2010; Klumpp & Hwa, 2014) (N Nikolic et al., 2017, unpublished data). Moreover, mCherry is a highly stable protein, thus the amount of mCherry fluorescence reflects mCherry production and dilution through cell division. As growth is impeded during adverse conditions (and mCherry is not diluted through cell division), and some stressful conditions additionally impair translation, these global factors would collectively affect the mCherry fluorescence signal.

Measurements of the entire bacterial populations, without gating, showed a high degree of variation in the constitutive *mCherry* expression during stressful conditions (Figs.  2F and 2G), which can signify heterogeneity in cell size, viability, and growth rate. The variation in mCherry fluorescence was highest during and after treatments with high concentrations of nalidixic acid (Fig. 2G), or ampicillin (Table 1), suggesting that cell lysis and severe growth retardation increase population heterogeneity. In contrast, the variation in mCherry fluorescence was lowest in populations experiencing longer recovery periods, i.e., in cultures initially stressed and analyzed after overnight recovery (Fig. 2H, Tables 1 and 2).

Interestingly, we found no correlation between the GFP and mCherry signals in the analyzed populations after mild Spec-treatment (gated only based on size parameters, Fig.  2I), and the same trend is also found in other populations initially stressed and analyzed after overnight recovery (see File S1 for Source Data). If we assume that constitutive mCherry fluorescence is an indicator of growth (Leveau & Lindow, 2001; Klumpp, Zhang & Hwa, 2009; Scott et al., 2010; Klumpp & Hwa, 2014) (N Nikolic et al., 2017, unpublished data), and GFP fluorescence is an indicator of the *mazEF* transcription, this lack of correlation could imply that de-repression and activation of the *mazEF* transcription do not depend on the cellular growth rate. This result is in line with previous population-based studies that have shown that the expression of an unregulated gene is growth-rate dependent, whereas the expression of a negatively autoregulated gene is a growth-rate independent process (Klumpp, Zhang & Hwa, 2009; Scott et al., 2010).

### MazF-mediated mRNA processing monitored by *gfp* reporter gene fusions

The increasing evidence that TA systems might introduce phenotypic heterogeneity in genetically identical populations (Gerdes & Maisonneuve, 2012; Klumpp & Hwa, 2014) prompted us to study the impact of the MazF-mediated processing on the translation of the respective mRNAs (Vesper et al., 2011; Sauert et al., 2016). To this end, we selected the *grcA* transcript, which has been shown to be cleaved by MazF at two ACA sites closely upstream of the start codon (Vesper et al., 2011; Sauert et al., 2016). The toxin MazF can thus modify the *grcA* mRNA by keeping a short leader sequence or making the mRNA leaderless, which allows its selective translation by stress-ribosomes (Vesper et al., 2011). Hence, the *grcA* mRNA represents a *bona fide* model to investigate the alteration of gene expression by MazF-mediated mRNA processing.

**Figure 3:**
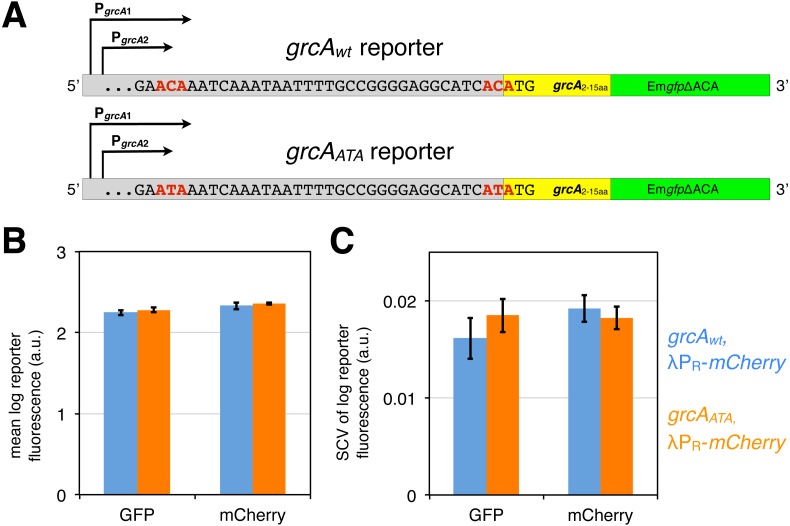
*grcA* reporters constructed to infer MazF-mediated mRNA processing. (A) The *grcA*_*wt*_ reporter comprises 278 nt upstream of the start codon of *grcA*, as well as the first 15 codons fused in frame to Em*gfp*ΔACA, which is a *gfp* variant devoid of ACA sites in the open reading frame (Sauert, 2015; Oron-Gottesman et al., 2016). The *grcA*_*ATA*_ reporter is a modified *grcA*_*wt*_ reporter harboring two nucleotide substitutions (C to T) at the positions previously determined to be processed by MazF (Vesper et al., 2011). (B) GFP and mCherry signals do not significantly differ between the *grcA*_*wt*_ and the *grcA*_*ATA*_ reporter measured in the exponential phase. Strains NN207 (depicted in blue) and NN214 (depicted in orange) harbor the *grcA*_*wt*_ and the *grcA*_*ATA*_ reporter, respectively, together with the constitutive *λP*_*R*_-*mCherry* reporter. (C) The differences are neither significant for the variation in GFP and mCherry fluorescence. Variation is defined as the squared coefficient of variation, SCV. We analyzed *N* = 5 independent replicates.

To follow the *grcA* expression pattern at the single-cell level, we constructed a fluorescent reporter gene comprising the 5′-UTR and the first 15 codons of *grcA* fused in frame to the *gfp* coding sequence, under control of the native *P*_*grcA*_ promoters (Fig. 3A). This wild-type reporter *grcA*_*wt*_ was used as a proxy for the impact of MazF-mediated mRNA processing on gene expression. As a control, we generated the *grcA*_*ATA*_ fluorescent reporter by substituting the ACA sequence motifs by ATA, to prevent MazF-mediated processing. In both reporters, we used an Emerald *gfp* version that does not contain ACA sites in the open reading frame (Em*gfp*ΔACA) (Sauert, 2015; Oron-Gottesman et al., 2016), while the amino acid sequence remains the same as in the wild-type Emerald GFP (Tsien, 1998).

First, we confirmed by flow cytometry that both, the *grcA*_*wt*_ and the *grcA*_*ATA*_ reporters display similar fluorescence signals during exponential growth without stressors, which would indicate that there are no significant differences in translation of the reporter mRNAs. Indeed, we observed no significant difference in the mean GFP fluorescence level between the *grcA*_*wt*_ and the *grcA*_*ATA*_ reporter (Fig. 3B, Fig. S1). Likewise, there was no difference in the variation in GFP fluorescence, measured as squared coefficient of variation (SCV, Fig. 3C). Alongside, we measured constitutive *mCherry* expression from the reporter *λP*_R_-*mCherry*. Correspondingly, we observed no significant differences in the mean level and variation in the mCherry fluorescence in strains harboring the different *grcA* reporters (Figs. 3B and 3C).

### Heterogeneity in the *grcA*_*wt*_ reporter fluorescence during recovery from *mazF* expression

Next, we aimed to investigate differences in fluorescence between the *grcA*_*wt*_ and *grcA*_*ATA*_ reporters in conditions that promote MazF activation. Because our fluorescent system is based on the GFP translation and subsequent fluorescence, and most of the known inducers of MazF activation are translational inhibitors (Sat et al., 2001; Christensen et al., 2003; Hazan, Sat & Engelberg-Kulka, 2004), we opted for an experimental setup in which *mazF* expression is tightly controlled by a synthetic promoter. Thus, we employed a chromosomally integrated system to express *mazF* under transcriptional control of the arabinose-inducible promoter *P*_BAD_ in order to investigate direct effects of MazF activation on translation of reporter mRNAs (sequence *P*_BAD_-*mazF* taken from the plasmid-based system pBAD-*mazF* (Amitai, Yassin & Engelberg-Kulka, 2004) (N Nikolic et al., 2017, unpublished data)). In this setup, *mazF* expression was ectopically induced at low levels (Fig. S2), as our results suggested that MazF is transcribed at low levels during antibiotic treatments or amino acid starvation (Tables 1 and 2).

**Figure 4:**
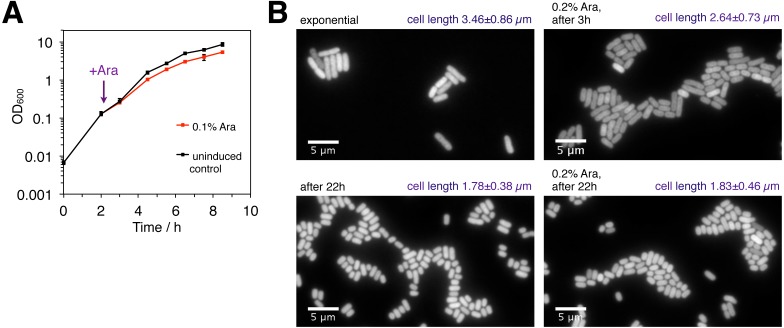
Effect of the mild MazF stress on bacterial growth and cell length. (A) Exponentially growing cultures of strain NN204 were divided into two flasks: 0.1% Ara was added to the first flask to ectopically induce *mazF* expression from the chromosomally integrated *P*_BAD_-*mazF* system, and the second flask served as a control. The maximum bacterial growth rate was measured as the slope of ln-transformed OD_600_ data measured at *t*_1_ = 3 h and *t*_2_ = 4.5 h, and the final OD_600_ was measured at *t* = 8.5 h. Here depicted are average values of *N* = 3 independent replicates; error bars present standard deviation. (B) An exponentially growing culture harboring the *λP*_*R*_-*mCherry* reporter and the chromosomally integrated system *P*_BAD_-*mazF* (strain NN207) was split in two flasks; 0.2% Ara was added to one flask, and the second flask served as the uninduced control. The samples were taken and cells were fixed after 3 h and 22 h. mCherry images were acquired with an epifluorescence microscope to infer variation in the cell length, indicated as mean ± standard deviation.

The analysis was performed using derivatives of the *E. coli* strain BW27784 (Khlebnikov et al., 2001). Strain BW27784 is suitable for arabinose-inducible expression systems such as *P*_BAD_-*mazF* because it constitutively takes up L-arabinose but is devoid of L-arabinose metabolism (Fig. S2). *mazF* expression was ectopically induced by addition of 0.1% arabinose (Ara) to exponentially growing cultures. It is important to note that bacterial growth did not cease under these conditions. This low level of ectopic *mazF* expression led to a 20% reduction in the bacterial growth rate and a decrease in the final optical density (OD_600_) by 36% (Fig. 4A). In contrast to previous observations that ectopic toxin overexpression causes cell elongation (Kasari et al., 2010), the mild ectopic *mazF* expression used here did not result in the formation of filamentous cells (Fig. 4B; all values in File S1).

**Figure 5:**
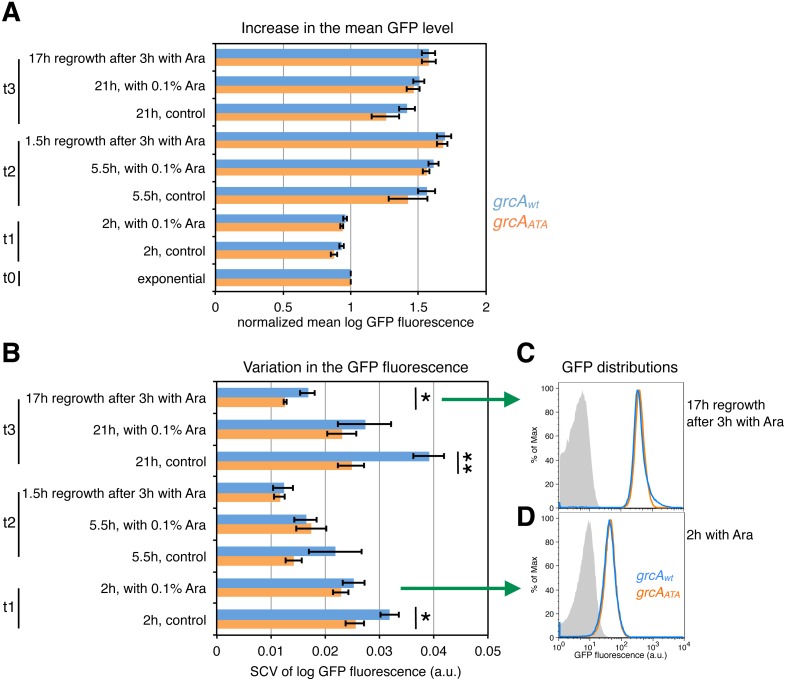
Effect of ectopic *mazF* expression on translation of the *grcA*_*wt*_ and * grcA*_*ATA*_ reporters. Fluorescence was measured at different time points: during exponential phase before *mazF* expression (t0), and 2 h (t1), 5.5 h (t2), and 21 h (t3) without or with addition of 0.1% Ara to ectopically induce *mazF* expression. In addition, we analyzed cultures that were washed 3 h after arabinose induction and recovered for 1.5 or 17 h in fresh media, respectively. (A) For analysis of the mean level of GFP fluorescence, the mean of each replicate was divided by the mean GFP fluorescence of the respective culture in the exponential phase, and referred to as ‘normalized mean’. The normalized mean level of log_10_-transformed GFP fluorescence was not significantly different between strain NN206 harboring the *grcA*_*wt*_ reporter (blue) and strain NN212 harboring the *grcA*_*ATA*_ reporter (orange). (B) Variation in the GFP fluorescence (SCV) was significantly higher in the *grcA*_*wt*_ reporter than in the *grcA*_*ATA*_ reporter in the overnight recovery phase after 3 h of stress caused by ectopic *mazF* expression (time point t3), and throughout the growth curve without added Ara. Significant *p*-values of *t*-tests are depicted (*p < 0.05, **p < 0.01). All *p*-values are listed in File S1. (C) The GFP fluorescence distribution is skewed towards higher values for *grcA*_*wt*_ reporter in the overnight recovery phase after 3 h of ectopic *mazF* expression (blue). The histogram of the *grcA*_*ATA*_ reporter is depicted in orange, and the reporterless strain NN204 is depicted in grey as the background level of GFP fluorescence, i.e., bacterial autofluorescence. The average value of skewness (kurtosis) is 0.023 (26.50) for the *grcA*_*wt*_ reporter and −0.227 (42.37) for the *grcA*_*ATA*_ reporter, measured in *N* = 3 replicates. The differences between the reporters are significant (*p* = 0.003 for skewness, *p* = 0.00098 for kurtosis), and all skewness and kurtosis values are reported in File S1. Here, the histograms of one replicate per strain are plotted. (D) For comparison, the GFP fluorescence distributions are not significantly different between *grcA*_*wt*_ and *grcA*_*ATA*_ reporters after 2 h of ectopic *mazF* expression.

After arabinose induction of *mazF* expression and during the recovery period after the removal of the inducer, we analyzed the increase in the mean level of GFP fluorescence encoded by the *grcA*_*wt*_ and the *grcA*_*ATA*_ reporters. We calculated normalized mean GFP fluorescence as the mean GFP level of each sample divided by the mean GFP level of the respective culture measured in the exponential phase. Despite the normalized mean levels of GFP fluorescence were similar between the *grcA* reporters (Fig. 5A), there were significant differences in variation in the GFP fluorescence (Fig. 5B). Interestingly, variation in the *grcA*_*wt*_ reporter fluorescence was higher than the *grcA*_*ATA*_ reporter fluorescence 17 h after recovery from 3 h of ectopic *mazF* expression. Likewise, we observed increased variation when cultures experienced nutrient deprivation during the overnight growth (stationary phase), without addition of the inducer (‘control’, time point t3 in Fig. 5B). Moreover, variation in the *grcA*_*wt*_ reporter fluorescence after overnight growth was significantly higher in the wild-type strain MG1655 containing native *mazEF* operon compared to the Δ*mazF* background (Fig. S3). These results suggest that conditions that promote MazF activation increase heterogeneity in the translation of the reporter mRNA that is processed by MazF, compared to the reporter mRNA that is not affected by MazF cleavage.

To further understand variable *gfp* expression within clonal populations during ectopic *mazF* expression, we employed two additional measures of variation, namely skewness and kurtosis, which signify the degree of asymmetry and the “tailedness” of GFP fluorescence distributions. Quantitative analyses of skewness and kurtosis showed statistically significant differences (*p* < 0.01) between the *grcA* reporters only for the fluorescence measurements 17 h after recovery from 3 h of ectopic *mazF* expression (see File S1 for Source Data). Interestingly, the corresponding histograms of GFP fluorescence revealed that the *grcA*_*wt*_ reporter GFP distributions had a right tail (Fig. 5C), i.e., the distributions were skewed toward higher values (for comparison see Fig. 5D). Specifically, GFP distributions of the *grcA*_*wt*_ reporter contained more highly fluorescent cells as compared to the *grcA*_*ATA*_ GFP distributions. This result is indicative of the generation of a small subpopulation of cells with an increased level of reporter gene expression as a consequence of the MazF-mediated processing of the *grcA*_*wt*_ mRNA. It has been previously established that stress response promotes functional ribosome heterogeneity (Byrgazov, Vesper & Moll, 2013; Starosta et al., 2014), and that during MazF-induced stress the total ribosome pool is comprised of canonical ribosomes as well as stress-ribosomes that are able to translate MazF-processed mRNAs (Vesper et al., 2011; Sauert et al., 2016). Thus, one plausible explanation is that during ectopic *mazF* expression both, the un-processed and processed forms of the *grcA*_*wt*_ reporter mRNA can be translated by canonical and stress-ribosomes, respectively, whereas the *grcA*_*ATA*_ reporter mRNA can solely be translated by canonical ribosomes that comprise one part of the total ribosome pool. Given that MazF processing affects more than 300 mRNA species in *E. coli* by trimming their 5′-UTRs various lengths (Sauert et al., 2016), it is feasible that processed mRNAs maintain different translational regulatory elements that influence their translation in a specific manner. To generally investigate translational heterogeneity of other MazF-processed mRNAs, a reporter system similar to the *grcA* reporter could be used. However, the reporter sequence has to be carefully optimized and adapted to each candidate mRNA, since we already observed differences in the reporter fluorescence between the wild-type and the ΔACA reporter prior to MazF stress for another MazF-processed mRNA, namely the *rpsU* mRNA (Fig. S4).

## Discussion

In this study we investigated how de-repression and transcriptional activation of the *mazEF* module differ between cells within a clonal population during and after antibiotic treatment, and nutritional stress. In all our experiments, we employed fluorescent reporters that are highly stable systems, and thus do not provide information on current physiological state (e.g., transient bursts in *mazEF* transcription) but rather describe cumulative changes in reporter gene expression between two flow cytometry measurements. The transcriptional reporter *P*_*maz*_-*gfp* was not expressed during exponential growth, indicating strong repression of the *mazEF* transcription in conditions without stress (Marianovsky et al., 2001; Christensen et al., 2003). During stressful conditions and in the recovery phase upon removal of the stressor, we measured an increase in the GFP fluorescence of bacterial populations indicating de-repression of the *mazEF* transcription. Interestingly, the reporter *P*_*maz*_-*gfp* was not expressed above background level in all analyzed cells within a population, which suggests that the *mazEF* module is transcribed at a low level during adverse conditions. A recent analysis of available transcriptome data has shown non-significant or minor changes in the *mazEF* mRNA level in response to stressful conditions that have not been addressed in this study, namely, treatment with tetracycline and rifampicin, starvation in 48 h-cultures, and heat shock (Muthuramalingam, White & Bourne, 2016). Another study measuring fluorescence of plasmid-based transcriptional reporter systems for TA modules indicated that the *mazEF* module is significantly induced by isoleucine starvation, osmotic stress, and phosphate starvation, albeit not continuously during the entire stress periods (Shan et al., 2017).

We also found no direct link between the fluorescence of the *P*_*maz*_-*gfp* reporter and constitutive mCherry fluorescence that served as an indicator of growth. *mazEF* expression is negatively autoregulated at the transcriptional level (Marianovsky et al., 2001; Keseler et al., 2013), and it has been previously suggested that expression of negatively autoregulated genes does not depend on the bacterial growth rate (Klumpp, Zhang & Hwa, 2009; Scott et al., 2010). Additionally, stress response mechanisms and activation of other TA systems (Gerdes & Maisonneuve, 2012; Kasari et al., 2013) might have additional roles in the regulation of *mazEF* expression.

Furthermore, it has been previously shown that de-repression of the *mazEF* transcription occurs when the ratio of toxin to antitoxin is in favor of the MazF toxin (Aizenman, Engelberg-Kulka & Glaser, 1996; Maisonneuve et al., 2011; Cataudella et al., 2013; Gelens et al., 2013). Hence, the reporter *P*_*maz*_-*gfp* could be employed to assess the level of temporarily unbound MazF that exerts its endoribonucleolytic activity. Here we report on a slight increase in the GFP fluorescence of the *P*_*maz*_-*gfp* reporter, which might also indicate increased levels of active MazF during stressful conditions and upon stress relief. Since an increased GFP signal implies elevated levels of *mazEF* transcription, the concentration of free MazF possibly increases only transiently before *de novo* production of both MazE and MazF. Thus, plasmid-based systems frequently employed to ectopically induce *mazF* overexpression (Vazquez-Laslop, Lee & Neyfakh, 2006) might generate levels of MazF activation that are considerably higher than herein measured during antibiotic treatments and nutritional stress.

Our data also highlight the impact of MazF-mediated mRNA processing on reporter gene expression. Interestingly, despite the population displays comparable mean fluorescence levels, the ectopic induction of *mazF* expression resulted in a higher variation in the *grcA*_*wt*_ reporter fluorescence when compared to the variation in the *grcA*_*ATA*_ reporter fluorescence. This higher variation can be attributed to increased GFP signal in a subpopulation of cells, indicating the increased *grcA*_*wt*_ reporter gene expression in those cells. This result suggests that the ability to translate both, the canonical and processed forms of *grcA*_*wt*_ reporter mRNAs by canonical and stress-ribosomes, respectively (Vesper et al., 2011; Byrgazov, Vesper & Moll, 2013), can lead to enhanced reporter protein synthesis in a subpopulation of cells.

A recent study has identified 330 MazF-processed, polysome-associated mRNAs that harbor 5′-UTRs of various lengths (Sauert et al., 2016). For instance, one form of the MazF-processed *grcA* transcript comprises 2 nucleotides upstream of the start codon, whereas the MazF-processed *rpoS* transcript comprises 108 nucleotides upstream of the start codon. In the case of the *rpoS* transcript, MazF-mediated cleavage removes only a part of the translational regulatory elements (Keseler et al., 2013), thereby possibly affecting *rpoS* expression dynamics in the conditions that promote MazF activation. Thus, it is feasible that MazF processing promotes a variety of changes in translational properties of the rendered mRNAs.

Taken together, our results suggest that MazF activation has a subtle effect in stressed bacterial populations during antibiotic treatment or amino acid starvation, which may further cross-activate other TA systems (Kasari et al., 2013; Wessner et al., 2015) or stress response mechanisms (Wang & Wood, 2011), collectively affecting bacterial gene expression and growth. A two-tier manifestation of cell-to-cell heterogeneity potentially occurs during the MazF-mediated stress response: first, the activation of the *mazEF* transcription is generally maintained at low levels, and the overall amount of MazF may be variable between single cells. Second, it is conceivable that mRNA processing by MazF fosters the variation in gene expression. Hence, we hypothesize that the heterogeneous MazF-mediated processing of mRNAs and rRNAs, and the modification of ribosomes to be selective towards translation of a small subset of MazF-processed mRNAs, could produce a variety of gene expression programs within isogenic populations. The variation in translation of MazF-processed mRNAs could depend on the specific regulatory regions that remained parts of the 5′-UTRs after MazF cleavage. In conclusion, populations may exhibit a substantial level of variation in gene expression during stressful conditions that trigger MazF and during resumption of growth upon stress relief.

##  Supplemental Information

10.7717/peerj.3830/supp-1 Supplemental Information 1 Supplementary Figures Figure S1. Fluorescence of the *grcA* reporters integrated in strains MG1655 and the isogenic *mazF* deletion mutant; Figure S2. The chromosomally integrated *P*_BAD_ expression system is inducible; Figure S3. Increased variation in the *grcA*_*wt*_ reporter fluorescence after overnight growth; Figure S4. *rpsU* reporters constructed to infer MazF-mediated mRNA processing.

10.7717/peerj.3830/supp-2 Table S1 List of strains and plasmids used in this study

10.7717/peerj.3830/supp-3 File S1 Supplementary File S1 –Source Data Datasets: source data, statistical tests, sequences, primers, R commands.

## Additional Information and Declarations

### Data Availability

The following information was supplied regarding data availability: All raw flow cytometry files were deposited to the FlowRepository (http://flowrepository.org) with assigned Repository IDs: FR-FCM-ZY93 (data for Fig. 1), FR-FCM-ZY9Y (Table 1), FR-FCM-ZY92 (Table 2), FR-FCM-ZY94 (Fig. 2I), FR-FCM-ZY95 (Fig. 3), FR-FCM-ZY96 (Figs. 5 and S2), FR-FCM-ZY98 (Fig. S1), FR-FCM-ZY99 (Fig. S3), and FR-FCM-ZY97 (Fig. S4).
