# Supplementary material for: MazF activation promotes translational heterogeneity of the grcA mRNA in Escherichia coli populations
Source: PeerJ. 2017 Sep 21;5:e3830. doi: 10.7717/peerj.3830 (PMC5610899; doi:10.7717/peerj.3830)
Supplement: Supplemental Information 1 — Figure S1. Fluorescence of the grcA reporters integrated in strains MG1655 and the isogenic mazF deletion mutant; Figure S2. The chromosomally integrated PBAD expression system is inducible; Figure S3. Increased variation in the grcAwt reporter fluorescence after overnight growth; Figure S4. rpsU reporters constructed to infer MazF-mediated mRNA processing. [file peerj-05-3830-s001.pdf]

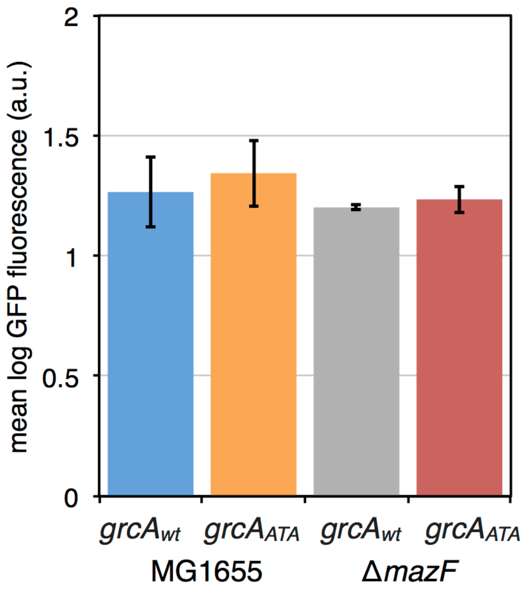

Figure S1. Fluorescence of the *grcA* reporters integrated in strains MG1655 and the isogenic *mazF* deletion mutant

We observed no significant difference in the mean level of GFP fluorescence when encoded by the $grcA_{wt}$ and the $grcA_{ATA}$ reporter gene in strain MG1655 (strains NN200 and NN211, respectively), and the isogenic *mazF* deletion strain (Müller et al., 2016) (strains NN208 and NN221); ANOVA with Post Hoc Test Bonferroni, p-values> 0.2; see Supplementary File S1. The analysis of N= 3 replicates was performed in the exponential phase, without any stressors. Error bars present standard deviation.

Although we used the same settings for all flow cytometry experiments, the experiments were conducted over a longer period of time; therefore different experiments may produce different absolute fluorescence values due to, for instance, repair and maintenance of the flow cytometers. We thus quantitatively compare only measurements conducted on the same day with the same flow cytometer, which we qualitatively compare with experiments conducted on a different day (e.g. mean GFP fluorescence levels in Figure 3B and Figure S1).

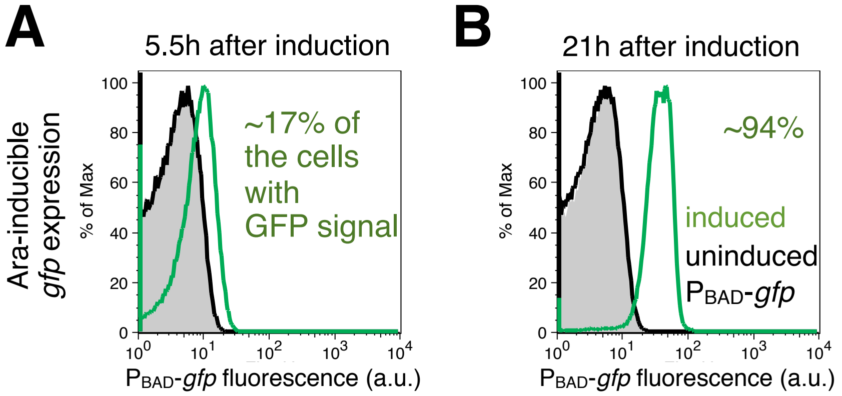

Figure S2. The chromosomally integrated P_{BAD} expression system is inducible

As a control experiment to quantify the Ara-inducible expression of a gene from the chromosomally integrated system, we replaced the *mazF* gene in the P_{BAD}-*mazF* expression system with the Em*gfp*ΔACA gene (resulting in strain NN209), and quantified the level of GFP fluorescence that can be produced when P_{BAD}-system is ectopically induced with 0.1% Ara. The measurements of the reporterless strain NN204 are depicted in grey, representing the background level of GFP fluorescence, i.e. bacterial autofluorescence. **A)** The GFP signal from the chromosomally encoded inducible system P_{BAD}-*gfp* was detected in 17% of the population after 5.5 hours of induction. Ectopic gene expression by arabinose induction is thus within the similar range as *mazEF* de-repression under stress conditions presented in Figure 2A. **B)** After 21 hours of induction with 0.1% arabinose, 94% of the population expressed *gfp* above background.

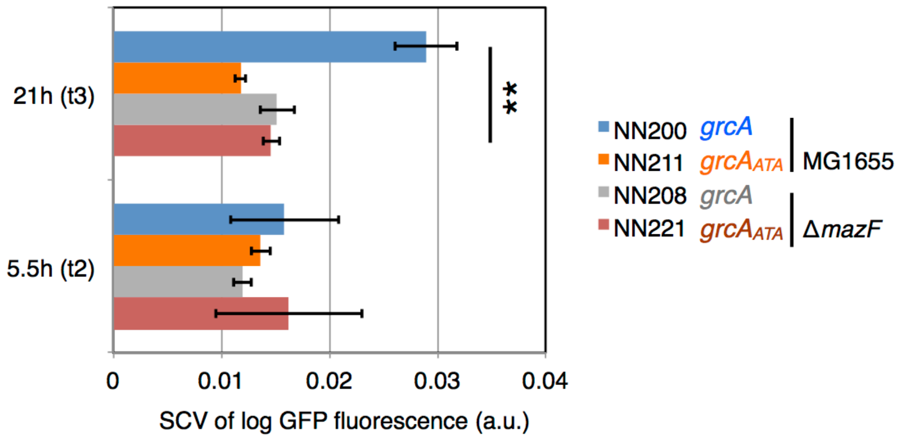

Figure S3. Increased variation in the *grcA*$_{wt}$ reporter fluorescence after overnight growth

GFP fluorescence was measured in the MG1655 strain (strains NN200 and NN211, respectively), and in the isogenic Δ*mazF* strain (Müller et al., 2016) (strains NN208 and NN221) during growth in defined rich media, without addition of the inducer of *mazF* expression. After overnight growth (time point t3), variation in the *grcA*$_{wt}$ reporter fluorescence (measured as squared coefficient of variation, SCV) was significantly higher in the wild-type MG1655 strain containing native *mazEF* operon (blue), compared to the isogenic Δ*mazF* strain (grey), as well as compared to the variation in the *grcA*$_{ATA}$ reporter fluorescence in the wild-type MG1655 strain (orange) and the isogenic Δ*mazF* strain (dark red) (** $p < 0.01$, ANOVA with Post Hoc Test Bonferroni, $p = 0.000036$; all p-values in Supplementary File S1). Error bars present standard deviation, N= 3 replicates.

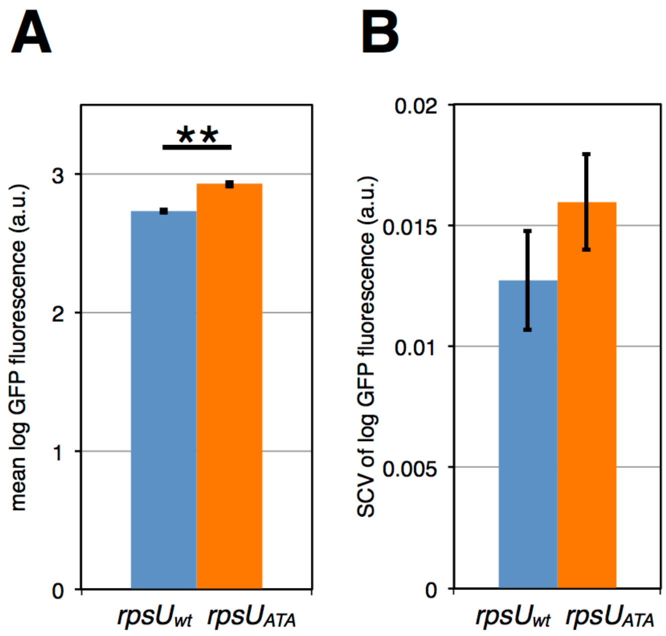

Figure S4. *rpsU* reporters constructed to infer MazF-mediated mRNA processing

The MazF toxin cleaves the *rpsU* transcript closely upstream of the start codon (Vesper et al., 2011). We thus attempted to develop an *rpsU* reporter system in addition to the *grcA* reporter system. The $rpsU_{wt}$ and $rpsU_{ATA}$ reporter-strains harbor the respective *gfp* reporter fusion comprising the transcriptional and translational regulatory regions of the *rpsU* gene (see Supplementary File S1 for sequences of the constructs).

A) The mean GFP fluorescence significantly differs between the $rpsU_{wt}$ and the $rpsU_{ATA}$ reporter measured in the exponential phase (** p< 0.01, 2-tailed *t*-test, p= 0.00034). Strains NN234 (depicted in blue) and NN236 (depicted in orange) harbor the $rpsU_{wt}$ and the $rpsU_{ATA}$ reporter, respectively. Error bars present standard deviation, N= 3 replicates. **B)** The difference in the variation in GFP fluorescence is not significant (2-tailed *t*-test, p= 0.181). Variation is defined as the squared coefficient of variation, SCV.

The difference in the mean GFP fluorescence suggests that there are significant differences in translation of the reporter mRNAs resulting from the mRNA sequence properties. As discussed in the main text, in order to determine specific MazF-mediated changes in mRNA translation, no intrinsic differences in the reporter fluorescence should be detected between the wild-type and the ΔACA reporter prior to MazF stress (as shown for the *grcA* reporters in Figure 3B and Figure S1). Therefore, the experimental framework has to be optimized separately for each investigated MazF-processed mRNA.
